# 

**Published:** 1850-01

**Authors:** 


### CONTENTS

OF NO. I.

### ORIGINAL COMMUNICATIONS.

ESSAYS AND CASES.

Art. I.—A Brief Account of the Epidemic Dysentery, as it occurred in Frankfort and Franklin county, Ky., during the summer and fall of 1849. By Wm. C. Sneed, M. D., of Frankfort, Ky. - - - - - 1

Art. II.—Cases of Purpura. By B. F. Wendel, M. D., one of the Physicians to Emigrants' Hospital, Ward's Island, New York, - - - - - - 13

Art. III.—A Brief Sketch of the Life, Character, and Writings of William Charles Wells, M. D., F. R. S., &c., &c. An Address delivered before the Louisville Medical Society, December 7th, 1849. By Elisha Bartlett, M. D., Professor of the Theory and Practice of Medicine, in the University of Louisville, Ky. - 22

Art. IV.—Notes on a Case of Fatal Poisoning by Metallic Arsenic. By B. Silliman, Jr., M. D., Professor of Chemistry and Toxicology in the University of Louisville, and of Chemistry applied to the Arts, in Yale College, - - - - - - 49

REVIEWS.

Art. V—.A Treatise on the Diseases of the Bones. By Edward Stanley, F. R. S., President of the Royal College of Surgeons of England, and Surgeon to St. Bartholomew's Hospital, - - - - 58

Art. VI.—The Three Kinds of Cod-Liver Oil; comparatively considered, with reference to their chemical and therapeutic properties. By L. J. de Jongh, M. D., of the

Hague. Translated from the German, with an appendix and cases, by EDWARD CAREY, M. D. To which is added an article on the subject from "Dunglison on New Remedies," - - - - - - 86

ORIGINAL INTELLIGENCE.

Cui Bono? - - - - - - 90
Professor Bartlett's Address, - - - - - - 91
Thè Bandage in its Application to Fractures, &c., - - 92
A Practical Treatise on the Diseases of Children. By D. Francis Condie, M. D. - - - - - 92
The Reviewer of Professor Dudley's Surgical Essays, - - 92

